# Supplementary material for: Effects of Fermented Herbal Tea Residue on Serum Indices and Fecal Microorganisms of Chuanzhong Black Goats
Source: Microorganisms. 2022 Jun 16;10(6):1228. doi: 10.3390/microorganisms10061228 (PMC9228005; doi:10.3390/microorganisms10061228)
Supplement: Supplementary file 1 [file microorganisms-10-01228-s001.zip › microorganisms-1746068-supplementary.pdf]

Table S1. Beneficial substances in herbal tea.

Beneficial substances	Periodicals	References
alkaloids	Agricultural Sciences In China	Utilization of Chinese herbal feed additives in animal production
apigenin	Current Research in Food Science	Changes in polysaccharides structure and bioactivity during Mesona chinensis Benth storage
aliphatic acids	Phytochemistry Letters	New iridoids from the flowers of Plumeria rubra "Acutifolia"
carbohydrate	Agricultural Sciences In China	Utilization of Chinese herbal feed additives in animal production
campesterol	Current Research in Food Science	Changes in polysaccharides structure and bioactivity during Mesona chinensis Benth storage
fiber	Agricultural Sciences In China	Utilization of Chinese herbal feed additives in animal production
flavonoid	Current Research in Food Science	Changes in polysaccharides structure and bioactivity during Mesona chinensis Benth storage
iridoids	Phytochemistry Letters	New iridoids from the flowers of Plumeria rubra "Acutifolia"
lignans	Phytochemistry Letters	New iridoids from the flowers of Plumeria rubra "Acutifolia"
mineral	Agricultural Sciences In China	Utilization of Chinese herbal feed additives in animal production
monoterpenes	Journal of Food Science and Technology	Hemical composition and antimicrobial activities of volatile oil extracted from Chrysanthemum morifolium Ramat.
organic acids	Agricultural Sciences In China	Utilization of Chinese herbal feed additives in animal production
protein	Agricultural Sciences In China	Utilization of Chinese herbal feed additives in animal production
phenols	Agricultural Sciences In China	Utilization of Chinese herbal feed additives in animal production
polysaccharides	Agricultural Sciences In China	Utilization of Chinese herbal feed additives in animal production
saponins	Current Research in Food Science	Changes in polysaccharides structure and bioactivity during Mesona chinensis Benth storage
sesquiterpenes compounds	Journal of Food Science and Technology	Hemical composition and antimicrobial activities of volatile oil extracted from Chrysanthemum morifolium Ramat.
tannins	Agricultural Sciences In China	Utilization of Chinese herbal feed additives in animal production
vitamins	Agricultural Sciences In China	Utilization of Chinese herbal feed additives in animal production

Table S2. Primer amplification information.

gene	primer	Tm	product size
16sRNAV1-V9	Forwar：5'-AGAGTTTGATCCTGGCTCAG-3'<br>Reverse：5'-GNTACCTTGTTACGACTT-3'	55℃	1466bp

Table S3. PCR amplification information.

Number of Cycles	Temperature	Time
1 cycle	95℃	2 min
30-35 cycles	95℃	20 sec
	Tm-5℃	20 sec
	72℃	4 kb/min
1 cycle	72℃	5 min

Table S4. PCR system and conditions

Component	Volume	Final
Template	Variable	as required
Forward Primer (10 μM)	1 μl	0.2 μM
Reverse Primer (10 μM)	1 μl	0.2 μM
5×TransStar® FastPfu Buffer	10 μl	1×
2.5 mM dNTPs	4 μl	0.2 mM
5×TransStar® FastPfu DNA Polymerase	1 μl	2.5 units
Nuclease-free Water	Variable	-
Total volume	50 μl	-

Table S5. Effects of different proportion of FHTR on serum biochemical indices of Chuanzhong black goats.

Items	Treatment			SEM	*p*–value[a]	*p*–value[b]	
	CON	L15	L30		Diet* Time	line	quad
ALB, (g/L)							
0d	22.02	22.94	21.78	0.762		0.906	0.556
17d	21.48	22.14	22.00	0.724	0.217	0.790	0.813
35d	21.42	22.20	24.76	0.879		0.137	0.633
TP, (g/L)							
0d	65.78	64.84	63.24	1.388		0.494	0.917
17d	64.28	65.04	68.20	1.934	0.921	0.446	0.785
35d	67.08	66.52	71.94	1.795		0.294	0.450
GLOB, (g/L)							
0d	43.76	41.90	41.46	1.323		0.515	0.815
17d	42.80	42.90	46.20	1.943	0.910	0.511	0.719
35d	45.66	44.32	47.18	1.633		0.726	0.578
TC, (mmol/L)							
0d	1.55	1.62	1.65	0.090		0.693	0.932
17d	1.78	1.95	1.73	0.092	0.733	0.835	0.357
35d	1.55	1.55	1.44	0.088		0.646	0.775
UREA, (mmol/L)							
0d	6.12	4.99	4.71	0.393		0.159	0.616
17d	4.69	5.96	5.49	0.239	0.410	0.144	0.074
35d	5.39[a]	6.51[ab]	7.89[b]	0.491		0.038	0.894
NEFA, (mmol/L)							
0d	0.08	0.07	0.09	0.004		0.334	0.107
17d	0.09	0.08	0.08	0.005	0.079	0.262	0.776
35d	0.06	0.06	0.08	0.006		0.341	0.488
HDL-C, (mmol/L)							
0d	0.92	0.95	0.96	0.027		0.659	0.848
17d	1.04	1.14	1.05	0.039	0.568	0.984	0.311
35d	0.98	0.98	0.92	0.036		0.528	0.732
LDL-C, (mmol/L)							
0d	0.48	0.50	0.50	0.038		0.890	0.900
17d	0.49	0.50	0.48	0.039	0.501	0.925	0.922
35d	0.42	0.39	0.48	0.041		0.575	0.532
AST, (U/L)							
0d	79.68	84.88	85.24	4.250		0.625	0.805
17d	99.18	105.50	82.36	6.273	0.921	0.287	0.282
35d	88.30	103.76	78.34	5.803		0.473	0.105

AST, Aspartate aminotransferase; HDL–C, High-density lipoprotein-cholesterol; TP, Total protein; ALB, Albumin; TC, Total cholesterol; UREA, Urea; GLOB, Globulin; TG, Triglyceride; LDL–C, Low-density lipoprotein-cholesterol; NEFA, Non-esterified fatty acid; SEM, standard error of mean. Means with different letters in the same row (a–c) differ ($p < 0.05$), and no letters or the same letters are not significantly different. CON, 0% FHTR in the diet, L15, 15% FHTR in the diet; L30, 30% FHTR in the diet. *p*–value[a], interaction of feeding time and diet. *p*–value[b] represent p–values for linear and quadratic orthogonal contrasts for diet. *, interaction analysis.

Table S6. Effects of different proportion of FHTR on oxidative stress indices of Chuanzhong black goats.

Items	Treatment			SEM	*p*–value[a]	*p*–value[b]	
	CON	L15	L30		Diet* Time	line	quad
MDA, (nmol/mL)							
0d	21.71	20.18	20.77	0.646		0.579	0.474
17d	13.73	15.29	15.34	0.529	0.451	0.239	0.512
35d	15.34	14.74	14.22	0.669		0.530	0.980
GSH-Px,(U/mL)							
0d	105.24	110.84	101.04	6.397		0.945	0.677
17d	131.57	126.95	133.18	6.975	0.523	0.931	0.739
35d	136.79[a]	132.24[a]	159.61[b]	5.062		0.101	0.048
T-AOC, (U/mL)							
0d	0.39	0.35	0.35	0.022		0.492	0.719
17d	0.41	0.42	0.45	0.021	0.506	0.400	0.847
35d	0.57	0.49	0.52	0.025		0.425	0.342
OH-, (U/mL)							
0d	711.64	745.18	715.35	18.718		0.940	0.464
17d	636.13	599.93	613.03	18.650	0.245	0.640	0.566
35d	598.16[a]	625.25[a]	706.05[b]	17.838		0.008	0.384
BHBA, (μmol/L)							
0d	363.26	418.45	436.04	18.483		0.122	0.628
17d	383.17	407.62	385.70	10.589	0.863	0.927	0.340
35d	309.02	306.95	302.29	16.521		0.880	0.973

CRP, C reactive protein; BHBA, Beta-hydroxybutyric acid; OH−, Inhibition of hydroxyl radical; T−AOC, Total antioxidant capacity; MDA, Malonaldehyde; GSH-PX, Glutathione peroxidase. Means with different letters in the same row (a−c) differ ($p < 0.05$), and no letters or the same letters are not significantly different. CON, 0% FHTR in the diet, L15, 15% FHTR in the diet; L30, 30% FHTR in the diet. *p*−value[a], interaction of feeding time and diet. *p*−value[b] represent *p*−values for linear and quadratic orthogonal contrasts for diet.*, interaction analysis.

Table S7. Effects of different proportion of FHTR on immune indices of Chuanzhong black goats.

Items	Treatment			SEM	*p*– value[a]	*p*– value[b]	
	CON	L15	L30		Diet* Time	line	quad
IgM, (μg/mL)							
0d	2090.14	1969.08	2108.00	78.346		0.930	0.473
17d	2215.60	2166.52	1953.21	69.144	0.186	0.136	0.574
35d	2268.26	2316.12	2501.78	93.546		0.344	0.743
IgG, (mg/mL)							
0d	4.98	6.21	5.18	0.328		0.806	0.120
17d	6.19	6.18	5.96	0.331	0.761	0.792	0.895
35d	7.87	7.74	6.98	0.323		0.290	0.661
IgA, (μg/mL)							
0d	273.05	274.46	296.86	8.573		0.286	0.580
17d	323.50	306.10	325.94	13.505	0.967	0.946	0.553
35d	381.31	362.90	393.68	9.356		0.602	0.242
IL-2, (pg/mL)							
0d	685.47	708.37	765.85	50.182		0.550	0.881
17d	834.81	833.91	763.09	38.908	0.672	0.488	0.694
35d	1082.51	926.43	974.83	48.627		0.388	0.345
TNF-α, (pmol/L)							
0d	4.62	6.65	4.80	0.703		0.919	0.222
17d	5.88	5.43	5.72	0.680	0.972	0.929	0.815
35d	3.66	3.79	3.97	0.549		0.834	0.986

TNF−α, Tumor necrosis factor-alpha; IL−2, Interleukin-2; IgA, Immunoglobulin A; IgM, Immunoglobulin M (IgM); IgG, Immunoglobulin G. Means with different letters in the same row (a−c) differ ($p < 0.05$), and no letters or the same letters are not significantly different. CON, 0% FHTR in the diet, L15, 15% FHTR in the diet; L30, 30% FHTR in the diet. *p*−value[a], interaction of feeding time and diet. *p*−value[b] represent p-values for linear and quadratic orthogonal contrasts for diet. *, interaction analysis.

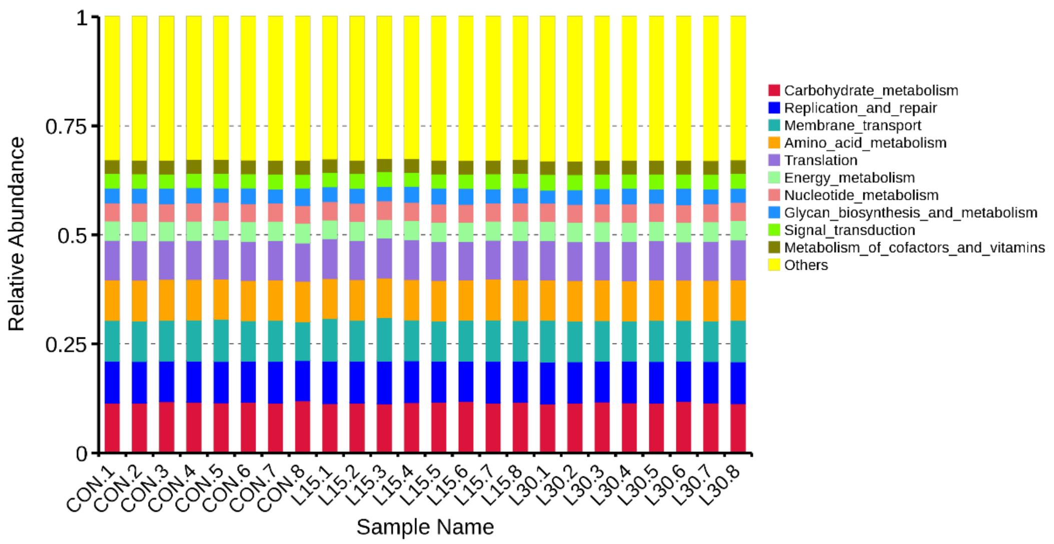

Figure S1. The top 10 predicted functions of level 2 of the fecal microorganism analyzed via Tax4fun. CON, 0% fermented herbal tea residue silage (FHTR) in the diet, L15, 15% (FHTR) in the diet; L30, 30% (FHTR) in the diet.
